# Guiding intensive care physicians’ communication and behavior towards bereaved relatives: study protocol for a cluster randomized controlled trial (COSMIC-EOL)

**DOI:** 10.1186/s13063-018-3084-7

**Published:** 2018-12-22

**Authors:** Nancy Kentish-Barnes, Sylvie Chevret, Elie Azoulay

**Affiliations:** 1 AP-HP, Saint Louis University Hospital, Medical Intensive Care Unit, Famiréa Group, 1 avenue Claude Vellefaux, Paris, France; 2 ECSTRA Team, Biostatistics and Clinical Epidemiology, UMR 1153 (CRESS), INSERM, Paris Diderot Sorbonne University, Paris, France

**Keywords:** Intensive care, End-of-life, Communication, Palliative care

## Abstract

**Background:**

Providing appropriate support and care for end-of-life patients and their relatives is a major concern and a daily responsibility for intensivists. Bereaved relatives of non-surviving patients in intensive care units (ICUs) often suffer from prolonged grief, posttraumatic stress disorder, anxiety, and depression. A physician-driven intervention, consisting of three meetings with the family, might reduce the post-ICU burden of bereaved family members 6 month after death. The patient’s nurse is actively involved at each step. We hypothesize that this strategy will improve communication in the end-of-life setting and thus, should reduce the post-ICU burden for family members, specifically the development of prolonged grief 6 months after the death.

**Methods/design:**

The COSMIC-EOL trial is a prospective, multicenter, cluster randomized controlled trial in which centers are allocated to two parallel arms: (1) intervention centers where relatives benefit from three-step physician-driven support during the dying and death process and (2) control centers where, during the dying and death process, relatives receive the standard of care practice. Each of the 36 participating centers will include 25 relatives of patients with a length of stay ≥2 days. Participating relatives will be followed up by phone at 1, 3, and 6 months after the patient’s death to complete questionnaires permitting evaluation of their post-ICU burden. The main outcome is prolonged grief measured 6 months after the death using the PG-13. Other outcomes include evaluation of quality of dying, quality of communication, anxiety, depression, and post-traumatic stress. The estimated duration of the study is 36 months.

**Discussion:**

The results of the trial will provide information about the effectiveness of physician-driven support for relatives of patients dying in an ICU. The study is expected to demonstrate a decrease in the ICU burden for bereaved relatives who benefitted from this intervention.

**Trial Registration:**

ClinicalTrials.gov, NCT02955992. Registered on November 3rd 2016.

**Electronic supplementary material:**

The online version of this article (10.1186/s13063-018-3084-7) contains supplementary material, which is available to authorized users.

## Background

### End-of-life and family burden

Providing appropriate support and care for end-of-life (EOL) patients and their relatives is a major concern for intensivists and, with a mortality rate of 20%, it has become a daily responsibility. Among those deaths, over 60% follow a decision to withhold or withdraw treatment [1]. In these situations, physicians, nurses, and relatives must work together towards the most consensual decision. In an intensive care unit (ICU), relatives are no longer simple visitors: they play active roles both at the patient’s bedside and with the team, thus creating a complex and unprecedented interaction. Caring for relatives is now considered one of the responsibilities of an ICU caregiver [2].

In this context, patients’ relatives feel vulnerable and, in the months that follow the death, they are at high risk of presenting symptoms that negatively affect their quality of life, such as anxiety, depression, posttraumatic stress disorder (PTSD) symptoms [3, 4], and complicated grief. In a recent study [1], 52% of relatives presented complicated grief symptoms 6 months after the patient’s death, a fivefold higher frequency compared with the general population [5, 6]. The post-ICU burden is very high in bereaved relatives and requires specific attention.

### The importance of communication

Many studies have shown that communication with caregivers is one of the most highly valued aspects of care [7–9]. It impacts family members’ experience during the patient’s stay and after the patient’s death. Communication perceived as inconsistent, unsatisfactory, or uncomforting is associated with a higher risk of presenting with post-ICU burden [10]. The risk of presenting with PTSD-related symptoms increases when bereaved relatives feel that information is incomplete [4]. Discordance between family members’ preferences for decision-making and their actual decision-making roles is associated with a higher risk of presenting with PTSD symptoms [11]. Bereaved relatives reporting poor quality of communication are more at risk of developing complicated grief and PTSD-related symptoms [1].

Communication is an essential component of family support and can be both verbal and nonverbal. The quality of information is crucial in the ICU context, but physicians’ and nurses’ attitudes and their ability to express empathy, comfort, and reassurance also significantly affect relatives’ experience. Indeed, relatives who experienced the physician’s attitude as non-comforting are at higher risk of developing a post-ICU burden or complicated grief [12]. Effective listening is an important aspect of communication. During family meetings, ICU physicians often miss opportunities to listen and respond to the needs of the next of kin [13]. In a study by Selph and colleagues, there was a significant positive correlation between the number of empathic statements made during family conferences and the degree of family satisfaction with communication [14].

### Support for family members

Support for family members is a core function of palliative care. The need to improve communication during the EOL process in the ICU context is recommended in palliative care and family-centered care guidelines [15–17]. Effective communication, empathetic attitudes, and personalized interactions with a patient’s family can improve the bereaved family's long-term psychological outcomes [18]. Rather than being considered as passive visitors, relatives are encouraged to become active partners in EOL decision-making and care, creating opportunities for empowerment.

Communication is important throughout the patient’s ICU stay, from their admission to their death. Moreoever, communication surrounding EOL is crucial. During this period, relatives undergo considerable distress. Their experience resembles a vortex [19]: a downward spiral of prognoses, difficult decisions, feelings of inadequacy, difficulties in saying good-bye, ending with the loss of the loved one despite the technical efforts of the ICU. Communication may be the most important factor in EOL care in ICUs [20].

### Three communication opportunities

Based on our experience, in the EOL process, there are three critical communication opportunities with relatives of patients dying after a decision to withhold or withdraw life-sustaining treatment. This randomized trial intends to target each of these three stages specifically.

First, there should be an interview to prepare the relatives for the patient’s imminent death and to elicit their understanding of the situation [21]. The death of a loved one can create feelings such as fear, relief, panic, or disbelief, and relatives need opportunities to express their feelings and emotions and to discuss the organization of the dying process (spiritual beliefs and needs, possible involvement in the patient’s care, and their presence at the time of death) [22–25]. Attentive listening is a key component of EOL interviews. In this study, this step is an extension of the VALUE approach [21, 26], updated by findings from recent research [1, 27].

The second opportunity occurs during the dying and death process. Dying after implementing a decision to withhold or withdraw treatment can take time, time during which relatives are often at the patient’s bedside. For families, being present during the dying period is a complex experience. Relatives seek intimacy but also need explanations and reassurance [8]. A room visit from the physician and from the nurse gives opportunities for families to express their feelings and ask specific questions concerning the following [15]: spiritual beliefs, tenets of palliative care, the expected time of death, and their possible role in the patient’s care [28]. Some relatives need guidance as to what is the best way to support the person who is dying. Caregivers can encourage the relative to touch the patient, to close the curtains, or to do whatever may be appropriate in that specific situation.

The third opportunity is a post-death encounter [29]. In a qualitative study by Nelson and colleagues, relatives who experienced clinicians as supportive during the ICU stay nonetheless perceived an abrupt and distressing shift at the time of death [8]. Many relatives often have questions regarding the patient’s stay, the patient’s illness, and the patient’s death. No answers to these questions can hinder the grieving process. Furthermore, during the patient’s stay, relatives develop relationships with the staff, physicians, and nurses, and need an opportunity to say good-bye to them. Closure with the team that cared for the patient permits grieving [30].

ICU caregivers have a great deal of responsibility in facilitating relatives’ involvement and in limiting their stress and difficulties. Results from various studies show that relatives’ satisfaction depends on the attitude of the caregivers as well as on good communication, good listening, and good information [31]. Communication at the EOL is a process that cannot be reduced to one interview. Providing multiple opportunities for relatives to ask questions, to express emotions, and to find comfort and support is an important aspect of palliative care.

In this randomized controlled trial, we will study efforts to integrate palliative care communication in intensive care more effectively and efficiently and will document any improvement using valid and responsive outcome measures.

### Meeting palliative needs in the ICU

There have been several studies suggesting that interventions to improve communication in the ICU could improve EOL care. To date, only very few intervention studies have examined patient- or family-centered outcomes. The first [21] focused on family conferences before the patient’s death. This positive randomized trial demonstrated that a proactive family conference and a bereavement pamphlet for relatives reduce post-traumatic stress symptoms 3 months after the death. However, 45% of relatives still presented with PTSD-related symptoms in the intervention group (compared to 69% in the control group), leaving space for more improvement.

The second [32] focused on clinician training. This negative cluster-randomized trial targeted five components of clinical care: clinician education, local champions, academic detailing, clinician feedback of quality data, and system supports. It showed no improvement in quality of dying and no change in ICU length of stay prior to death or time from ICU admission to withdrawal of life-sustaining measures. This shows that improving ICU EOL care requires interventions with more direct contact with patients and family, as in the previous study.

A third more recent study [33] showed that family informational and emotional support meetings led by palliative care clinicians does not improve family anxiety and depression; it may even increase PTSD symptoms. These findings do not support routine or mandatory palliative care-led discussion of the goals of care for all families of patients with a chronic critical illness.

For the first time, we propose a study with a direct ICU physician-driven intervention during the dying and death period, the most crucial time for family members. We designed a three-step physician-driven support strategy that consists of three meetings with the relative: one before, one during, and one after the patient’s death. The patient’s nurse is actively involved at each step.

### Hypothesis

We hypothesize that this strategy will improve communication in the EOL setting and thus, should reduce the post-ICU burden for family members, specifically the development of prolonged grief 6 months after the death.

## Methods/design

The COSMIC-EOL trial (Communication Strategies and Measures in Intensive Care at End of Life) is a prospective, multicenter, cluster randomized controlled trial. Centers are allocated to two parallel arms: (1) intervention centers where relatives benefit from three-step physician-driven support during the dying and death process and (2) control centers where, during the dying and death process, relatives receive the standard of care practice. This design was used to reduce the effect of intervention contamination, given that it involves training and education of health professionals to improve patient care.

### Objectives

This study aims to assess whether a three-step physician-driven support strategy during the dying and death process in the ICU can reduce patients’ relatives’ symptoms of prolonged grief 6 months after the patient’s death.

Secondary objectives are to evaluate in both groups of relatives: their experience of dying and death in the ICU; their satisfaction with EOL communication (specifically their rapport with the physician); and the impact on them of the three-step physician-driven support during the dying and death process on symptoms of anxiety, depression, and PTSD 1, 3, and 6 months after the death.

### Study setting

The study will take place at 36 ICUs in France (Additional file 1).

### Eligibility criteria

ICUs with ≥8 beds, a ratio of 1/2.5 nurses per patient, and 24 × 7 medical coverage were eligible. Participating centers were selected from (1) the Famiréa network, based on previous and repeated participation in Famiréa studies and (2) a list of centers that had never participated in Famiréa studies. Famiréa is a French research group that aims to study family members’ experience both during and after the patient’s ICU stay to improve practices, specifically communication skills and EOL care. Since its creation 20 years ago, it has promoted research into family-centered care in a network of ICUs that strive to put into practice the findings of published studies.

The study population consists of bereaved family members after the death of an adult patient in an ICU who fulfil all the inclusion criteria and none of the exclusion criteria.

Relatives of patients who meet the following criteria are included: patient >18 years who died in an ICU after a decision to withhold or withdraw treatment and ICU length of stay ≥2 days. To be included, relatives must be available to give consent to participate in the study. Relatives who do not understand, read, or speak French or who refuse to participate are excluded, as well as relatives of organ donor patients (as these relatives benefit from specific support and adapted communication by the transplant coordination teams).

As in previous Famiréa studies [1, 4, 21], only one family member is included. This family representative is the designated health-care proxy. In their absence, this is the family member most involved in the relationship with the ICU team, or by default is the spouse (or partner). In their absence, it is the parents or children of the patient, then another member of the family.

### Methodology

**Fig. 1:**
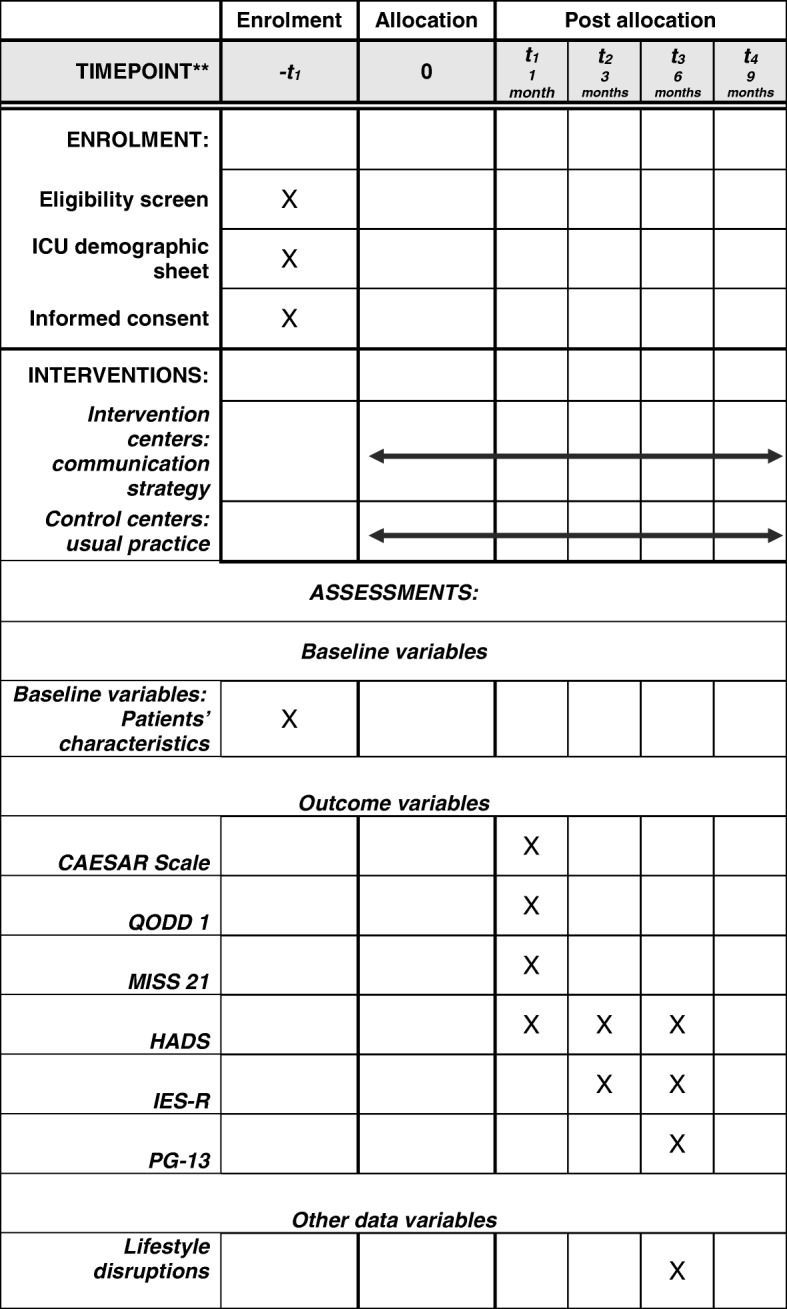
SPIRIT figure. HADS Hospital Anxiety and Depression Scale, ICU intensive care unit, IES-R Impact of Event Scale, Revised, MISS-21 Medical Interview Satisfaction Scale, PG-13 Prolonged Grief 13-item questionnaire, QODD-1 Quality of Dying and Death 1-item questionnaire

This protocol follows the Standard Protocol Items: recommendations for interventional trials (SPIRIT) checklist (Additional file 2). The SPIRIT recommended schematic diagram detailing the schedule of enrolment, interventions and assessments is provided as Fig. 1.

#### Randomization

**Fig. 2:**
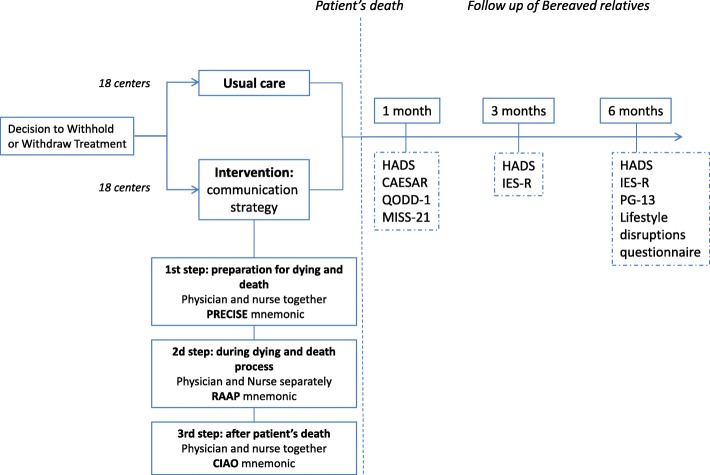
Study design

Participating centers are assigned by simple random allocation to one of the two groups in a 1:1 ratio. Each subject is treated according to the strategy allocated to the center (Fig. 2). The randomization between the two parallel groups of centers was centrally generated by a statistician not involved in the study using permuted and balanced blocks of non-released size. Randomization is stratified using recruitment period, and also on centers’ previous participation in Famiréa studies (experienced vs. new centers).

Relatives are included after the decision to withhold or withdraw treatment has been made using a signed consent form approved by the institutional review board. Relatives who consent to participate are contacted by phone three times: 1, 3, and 6 months after the patient’s death. All telephone calls are done by trained sociologists and psychologists from the Famiréa group. Telephone interviewers are blinded (control vs. intervention). The estimated duration of each call 1, 3, and 6 months after the patient’s death is 40 min, 30 min, and 30 min, respectively.

#### Study arms

Intervention pertains to the cluster and individual participant level.

In the control group, the bereaved relatives receive usual care during the dying and death process. In the intervention group, the bereaved relatives receive the three-step physician-driven support during the dying and death process. Relatives attending control or intervention centers are followed up by the Famiréa research group using telephone interviews 1, 3, and 6 months after the death to complete the questionnaires. This method has been validated in numerous follow-up studies of bereaved relatives [1, 4, 21].

The control centers manage the EOL process and communication with relatives using their standard of care. In these centers, EOL care is described for each patient in the patient’s characteristics form, which is used to record those who spontaneously benefited from a family conference, from a room visit during the dying and death process, or from a post-death meeting.

The intervention centers implement the three-step physician-driven support strategy. In these centers, prior to patient recruitment, the three-step communication strategy is presented to caregivers using video support. The team (physicians and nurses) then attend interactive educational meetings to set the importance of EOL communication. These meetings are led by members of the Famiréa research group and will cover themes such as family experience in the ICU and their experience of EOL, the impact of the ICU experience on bereaved relatives, communication in the EOL context, what families recall 1 year after the patient’s death, what caregivers can do to improve families’ experience and alleviate their burden, etc. All caregivers receive a leaflet about the importance of EOL communication. This lists the key elements (the impact of communication on a family’s post-ICU burden) and recommendations regarding verbal and nonverbal communication. As recommended in the literature on implementing guidelines [34–36], a local champion (or opinion leader) is designated by each team to help implement the strategy. An implementation phase of one month is required for the team to test and to get accustomed to the intervention as well as to discuss difficulties with the principal investigator.

Centers implement the three-step physician-driven support for a family after a decision to withhold or withdraw life-sustaining therapies, as shown in Table 1. The intervention is accompanied by a fidelity checklist to control adherence to the protocol.

**Table 1:** Study intervention: a three-step communication strategy

Three-step strategy	Preparation for death The physician and the nurse meet the relative to:	During the dying and death process:	After the patient’s death The physician and the nurse meet the relative to:
Themes	▪ Prepare the relative for the patient’s imminent death ▪ Provide an opportunity for relatives to ask questions ▪ Provide an opportunity for relatives to express their feelings and emotions ▪ Encourage relatives to talk to the patient and say good-bye ▪ Discuss being present at the time of death ▪ Discuss possible involvement in the patient’s physical care ▪ Discuss spiritual beliefs and needs	▪ The physician enters the patient’s room at least once to check whether the relatives have any questions or apprehensions and to check their comprehension of what is happening ▪ The nurse enters the patient’s room at least once to check the relatives’ comfort and needs, and whether they have any questions	▪ Express their condolences ▪ Elicit and answer questions about the patient’s death ▪ Provide an opportunity for relatives to express their feelings ▪ Offer them the option of contacting the team during their bereavement
Mnemonic	PRECISE: ▪ Prepare ▪ Relatives for the patient’s death ▪ Elicit questions and emotions ▪ Communication about presence at time of death and ▪ Involvement in care ▪ Spiritual needs ▪ Encourage relatives to say good-bye to the patient	RAAP: ▪ Reassure relatives regarding their spiritual beliefs, the tenets of palliative care, the expected time of death, and their role in the patient’s care ▪ Answer relatives’ questions ▪ Attentive listening ▪ Propose emotional support	CIAO: ▪ Condolences: express condolences ▪ Instigate questions about the patient’s ICU stay and death ▪ Acknowledge emotions ▪ Option to contact the team during their bereavement

In two of the intervention centers, an outsider observer (a sociologist) will observe clinical practices and meetings with the family members at steps 1, 2, and 3. The aims are (1) to complete the checklists independently and compare them with those completed by caregivers and (2) to observe nonverbal communication (voice, touch, and distance), such as the synchronization between nonverbal communication and emotions and caregivers’ capacity to adapt to each emotional situation.

#### Qualitative interviews

To evaluate the communication strategy and also to provide an in-depth comprehension of relatives’ experience of EOL communication in both arms (control and intervention), semi-structured interviews are organized voluntarily with relatives. The interviews are led by sociologists with extensive experience of research with bereaved relatives. Themes include: their memories of EOL care, their communication with physicians (verbal, nonverbal, and empathy), their communication with nurses (verbal, nonverbal, and empathy), their communication with the dying patient, time of death, post-death communication, and interaction with the ICU team.

#### Outcomes

Outcomes are at the individual patient level. The primary outcome is the Prolonged Grief 13-item questionnaire (PG-13) [37], completed 6 months after the patient’s death. The PG-13 measures five criteria for prolonged grief disorder: event (bereavement); separation distress; duration (i.e., >6 months since the bereavement); cognitive, emotional, and behavioral symptoms; and social or occupational impairment. The score range is 11–55 and a score of 36 or more is a clinical indicator of prolonged grief disorder. In addition, all five criteria must be met.

#### Secondary outcomes

The secondary outcomes are the following scales:

- CAESAR scale [38]: Experience of the patient’s dying and death (1 month after patient’s death). It determines the family member’s experience of EOL. There are three categories of responses:

- Difficult experience, low scores ≤59
- Moderate experience, scores ranging between 60 and 68
- Good experience, high scores ≥69

- QODD-1 – Quality of Dying and Death [39]: One question evaluating global satisfaction with the quality of dying and death (1 month after patient’s death)

- MISS-21 – Medical Interview Satisfaction Scale – the Rapport Subscale only (1 month after the patient’s death) [40]

- HADS – Hospital Anxiety and Depression Scale [41] (1, 3, and 6 months after the patient’s death): There are two subscales, anxiety and depression. Scores for each subscale range from 0 to 21, categorized as follows:

- normal 0–7
- mild 8–10
- moderate to severe 11–21

Scores for the entire scale (emotional distress) range from 0 to 42, with higher scores indicating more distress.

- IES-R – Impact of Event Scale, Revised [42]: Risk of presenting with PTSD symptoms (3 and 6 months after the patient’s death). It assesses the subjective distress caused by traumatic events. The total score ranges from 0 to 88. There are three categories of risk:

- Low risk, scores ranging between 1 and 11
- Moderate risk, scores ranging between 12 and 32
- High risk, scores of 33 and over

#### Exploratory measures

The following are also measured:

- Questionnaire (developed by the Famiréa group) for relatives about lifestyle disruptions (6 months)
- Qualitative semi-structured interviews with family members (9 months)
- Strategy checklist to assess adherence to the intervention (intervention centers only)
- Questionnaire for physicians and nurses in the intervention group to evaluate the communication strategy

In each center, the local investigator will complete an ICU characteristics form.

### Statistical analysis

The hypothesis is that the proactive communication strategy will decrease the proportion of relatives presenting complicated grief symptoms from 50% to 35% 6 months after the patient’s death (assumed decrease). To do so, we need to recruit 454 relatives (227 in each group) to give a power of 90% and a type I error rate α of 0.05. Moreover, given it is a cluster randomized trial, a between-cluster variation must be accounted for, using an inflation factor or design effect of $1+\left(\overline{n}-1\right)\uprho$, where $\overline{n}$ is the average cluster size and ρ is the intracluster correlation coefficient assuming the clusters are of a similar size [43]. Assuming a small clustering effect (intracluster correlation coefficient = 0.05), with an estimated average recruitment of 25 subjects per cluster, the inflation factor is set at 1.55, resulting in 704 enrolled subjects. As it is expected that 25% of the relatives will be lost to follow-up at 6 months [1, 4, 21], the sample size is increased to 874 (437 in each group).

The analysis will be done according to the intention-to-treat principle (each subject will be analyzed in the group to which they were was assigned by the randomization, regardless of whether or not it was effectively done). Descriptive statistics in each group (means, medians, or percentages as appropriate) will be used to assess any marked differences between the intervention and control groups. Point estimates with 95% confidence intervals will be reported for the primary outcome. This is a cluster randomized controlled trial, with correlation among patients from the same cluster.

The primary and secondary outcomes will be compared across randomized groups at the individual patient level, taking into account the correlation induced by the study design. The intracluster correlation coefficient or *k* statistic for each primary and secondary outcome will provide an indication of the extent of the clustering. Moreover, the correlation will be considered in the statistical analysis of the outcomes using mixed effects models, with both adjusted and unadjusted estimates [43, 44].

Missing data on covariates will use the multiple imputation by chained equation. The scores will be analyzed based on original data, and when available, according to established cutoffs. All tests will be bilateral and significance will be defined as *p* < 0.05.

### Additional analyses

The center effect will be tested. Intervention-by-subset interactions will be assessed to see whether the impact of the intervention is homogeneous or not in the three following predefined groups of relatives: being a spouse (vs. other relatives), French descent (vs. other origins), and aged over 65 years (vs. under 65). Also, a per protocol analysis will be performed for those relatives from the control group who unexpectantly received at least two of the three components of the intervention.

### Qualitative analysis

All interviews will be audio-taped and transcribed verbatim. All of the transcripts will be read extensively to gain an overall understanding of participants’ views and experiences. The data will then be analyzed thematically using the grounded theory methodology. Themes are not hypothesized prior to the interviews but will emerge from the respondents’ words. For the data analysis, we will use open, axial, and selective coding. In open coding, researchers categorize the data from the interview transcripts into broad categories or themes. In axial coding, each category is characterized and the relationships between categories are examined. Finally, in selective coding, the categories are organized into a framework to explain a phenomenon.

### Provisional timetable

Altogether, 36 centers (Additional file 1) are participating in the study. Based on the CAESAR study [1], four or five decisions to withhold or withdraw life-sustaining therapies are managed per month by each ICU. Each ICU will recruit 25 relatives. To permit a quality follow-up of the relatives and to avoid researchers being overloaded with follow-up telephone calls, relatives are enrolled in three time periods: centers 1 to 12 recruit during the first 6 months, centers 13 to 25 start during the next 6 months, and centers 26 to 36 during the final 6 months. Hence, recruitment is expected to last approximately 18 months. The follow-up of relatives will last for a total of 24 months.

### Strengths and limitations

The main difficulty in implementing this intervention aimed at improving communication and the attitudes of health-care professionals is adherence to the study protocol, since the strategy we propose concerns all members of the ICU team, including doctors and nurses. However, four strategies have been developed to limit the risk of non-adherence: (1) dissemination of a video presenting the strategy that can been seen at any time by any member of an intervention center, (2) quantitative monitoring of fidelity using a checklist for each inclusion, (3) qualitative monitoring in two ICUs by an outsider observer, and (4) regular interactions between the principal investigator and the local champions in each ICU to discuss difficulties and promote the correct implementation of the strategy.

Our study has several strengths: (1) it is a national study involving 36 ICUs, (2) it has a high number of inclusions, and (3) it is a strategy aimed at improving communication and behavior of doctors and nurses, thus increasing general awareness and changing the EOL culture.

### Data monitoring

The data will be monitored by the Clinical Research Unit of Saint Louis University Hospital. The data will be managed by the Biostatistics and Medical Information Service of Saint Louis University Hospital, Assistance Publique – Hôpitaux de Paris (AP-HP).

## Discussion

This study is focused on relatives of patients who died in an ICU. The psychological burden is extremely high for relatives in this context, with 50% presenting prolonged grief symptoms 6 months after the patient’s death, a prevalence that is 5 times higher than in the general population of bereaved relatives. Prolonged grief and posttraumatic stress symptoms considerably diminish quality of life. Prolonged grief is considered by some specialists as a distinct mental disorder on the grounds that it is a clinically significant form of psychological distress associated with substantial disability. The psychological burden co-occurs with lifestyle and employment disruptions as well as health problems. These will be measured in both groups of relatives so that we can understand any psychological, social, and economic benefits of the intervention.

Finding strategies to lower the psychological burden specific to the ICU setting is a necessity. If the study is positive, this communication strategy could be implemented in all French ICUs to reduce the burden, to help families during the grieving process, and to help reduce the social impacts of prolonged grieving. This intervention is costless and not difficult to implement. If the results are positive, this will be an extraordinary achievement. We will have shown how to reduce the burden during one of the most painful life experiences by improving EOL practices. Introducing this strategy to hospital units with high death rates would be an interesting objective.

### Trial status

The trial is currently recruiting relatives. Recruitment started on 23 February 2017.

## Additional files

Additional file 1: List of centers participating in the COSMIC-EOL trial. (DOCX 20 kb)

Additional file 2: SPIRIT 2013 Checklist: Recommended items to address in a clinical trial protocol and related documents. (DOC 121 kb)

## Abbreviations

AP-HP: Assistance Publique – Hôpitaux de Paris
COSMIC: Communication Strategies and Measures in Intensive Care
EOL: End-of-life
HADS: Hospital Anxiety and Depression Scale
ICU: Intensive care unit
IES-R: Impact of Event Scale, Revised
MISS-21: Medical Interview Satisfaction Scale
PG-13: Prolonged Grief 13-item questionnaire
PTSD: Posttraumatic stress disorder
QODD-1: Quality of Dying and Death 1-item questionnaire

## References

[1] Kentish-Barnes N, Chaize M, Seegers V (2015). Complicated grief after death of a relative in the intensive care unit. Eur Respir J.

[2] Aslakson RA, Curtis JR, Nelson JE (2014). The changing role of palliative care in the ICU. Crit Care Med.

[3] Pochard F, Darmon M, Fassier T (2005). Symptoms of anxiety and depression in family members of intensive care unit patients before discharge or death. A prospective multicenter study. J Crit Care.

[4] Azoulay E, Pochard F, Kentish-Barnes N (2005). Risk of Post-traumatic Stress Symptoms in Family Members of Intensive Care Unit Patients. Am J Respir Crit Care Med.

[5] Prigerson HG, Maciejewski PK, Peynolds CF (1995). Inventory of complicated grief: a scale to measure maladaptive symptoms of loss. Psychiatry Res.

[6] Kersting A, Brähler E, Glaesmer H, Wagner B (2011). Prevalence of complicated grief in a representative population-based sample. J Affect Disord.

[7] Nelson JE, Mulkerin CM, Adams LL (2006). Improving comfort and communication in the ICU: a practical new tool for palliative care performance measurement and feedback. Qual Saf Health Care.

[8] Nelson JE, Puntillo KA, Pronovost PJ, Walker AS, McAdam JL, Ilaoa D, Penrod J (2010). In their own words: patients and families define high-quality palliative care in the intensive care unit. Crit Care Med.

[9] Long AC, Curtis JR (2014). Quality of dying in the ICU: understanding ways to make it better. Intensive Care Med.

[10] Davidson JE, Jones C, Bienvenu OJ (2012). Family response to critical illness: post intensive care syndrome-family. Crit Care Med.

[11] Gries CJ, Engelberg R (2010). Predictors of symptoms of post traumatic stress and depression in family members after patient death in the ICU. Chest.

[12] Siegel MD, Hayes E, Vanderwerker LC (2008). Psychiatric illness in the next of kin of patients who die in the intensive care unit. Crit Care Med.

[13] Curtis JR, Engelberg RA, Wenrich MD (2005). Missed opportunities during family conferences about end-of-life care in the intensive care unit. Am J Respir Crit Care Med.

[14] Selph RB, Shiang J, Engelberg R (2008). Empathy and life support decisions in intensive care units. J Gen Intern Med.

[15] Nelson JE, Azoulay E, Curtis JR, Mosenthal AC, Mulkerin CM, Puntillo K, Siegel MD (2012). Palliative care in the ICU. J Palliat Med.

[16] Mularski RA, Osborne ML (2006). Palliative care and intensive care unit care: daily intensive care unit care plan checklist #123. J Palliat Med.

[17] Teno JM, Byock I, Field MJ (1999). Research agenda for developing measures to examine quality of care and quality of life of patients diagnosed with life-limiting illness. J Pain Symptom Manag.

[18] Davidson JE (2009). Family-Centered Care: Meeting the Needs of Patients’ Families and Helping Families Adapt to Critical Illness. Crit Care Nurse.

[19] Kirchhoff KT, Walker L, Hutton A (2002). The Vortex: Families’ Experiences With Death in the Intensive Care Unit. Am J Crit Care.

[20] Gauntlett R, Laws D (2008). Communication skills in critical care. Continuing Education in Anaesthesia. Crit Care Pain J.

[21] Lautrette A, Darmon M, Megarbane B (2007). A communication strategy and brochure for relatives of patients dying in the ICU. N Engl J Med.

[22] Curtis JR, White DB (2008). Practical Guidance for Evidence-Based ICU Family Conferences. Chest.

[23] Hudson PL, Aranda S, Kristjanson LJ (2004). Meeting the supportive needs of family caregivers in palliative care: challenges for health professionals. J Palliat Med.

[24] Hudson P, Quinn K, OHanlon B, Aranda S (2008). Family meetings in palliative care: Multidisciplinary clinical practice guidelines. BMC Palliat Care.

[25] Hudson P, Thomas T, Quinn K, Aranda S (2009). Family meetings in palliative care: are they effective?. Palliat Med.

[26] Curtis JR, Patrick DL, Shannon SE (2001). The family conference as a focus to improve communication about end-of-life care in the intensive care unit: opportunities for improvement. Crit Care Med.

[27] Kentish-Barnes N, McAdam JL, Kouki S (2015). Research Participation for Bereaved Family Members: Experience and Insights From a Qualitative Study. Crit Care Med.

[28] Puntillo K, Nelson JE, Weissman D (2014). Palliative care in the ICU: relief of pain, dyspnea, and thirst--a report from the IPAL-ICU Advisory Board. Intensive Care Med.

[29] Steinhauser KE, Voils CI, Bosworth H, Tulsky JA (2014). What constitutes quality of family experience at the end of life? Perspectives from family members of patients who died in the hospital. Palliat Support Care.

[30] Kentish-Barnes N, Prigerson HG. Is this bereaved relative at risk of prolonged grief? Intensive Care Med. 2016;42(8):1279–81. 10.1007/s00134-015-4182-6 26699916

[31] Andershed B (2006). Relatives in end-of-life care--part 1: a systematic review of the literature the five last years, January 1999-February 2004. J Clin Nurs.

[32] Curtis JR, Nielsen EL, Treece PD (2011). Effect of a quality-improvement intervention on end-of-life care in the intensive care unit: a randomized trial. Am J Respir Crit Care Med.

[33] Carson SS, Cox CE, Wallenstein S (2016). Effect of Palliative Care-Led Meetings for Families of Patients With Chronic Critical Illness: A Randomized Clinical Trial. JAMA.

[34] Borbas C, Morris N, McLaughlin B (2000). The role of clinical opinion leaders in guideline implementation and quality improvement. Chest.

[35] Bero LA, Grilli R, Grimshaw JM (1998). Closing the gap between research and practice : overview of systematic reviews of interventions to promote the implementation of research findings. BMJ.

[36] Davis DA, Taylor-Vaisey A (1997). Translating guidelines into practice. Can Med Assoc.

[37] Prigerson HG, Horowitz MJ, Jacobs SC (2009). Prolonged grief disorder: Psychometric validation of criteria proposed for DSM-V and ICD-11. PLoS Med.

[38] Kentish-Barnes N, Seegers V, Legriel S (2016). CAESAR: a new tool to assess relatives' experience of dying and death in the ICU. Intensive Care Med.

[39] Curtis JR, Patrick DL, Engelberg RA (2002). A measure of the quality of dying and death. Initial validation using after-death interviews with family members. J Pain Symptom Manag.

[40] Meakin R, Weinman J (2002). The 'Medical Interview Satisfaction Scale' (MISS-21) adapted for British general practice. Fam Pract.

[41] Zigmond AS, Snaith RP (1983). The hospital anxiety and depression scale. Acta Psychiatr Scand.

[42] Weiss DS, Marmar CR, Wilson JP, Keane TM (1997). The impact of event scale – revised. Assessing psychological trauma and PTSD.

[43] Campbell MK (2004). CONSORT statement: extension to cluster randomised trials. BMJ.

[44] Donner A, Klar N (2004). Pitfalls of and Controversies in Cluster Randomization Trials. Am J Public Health.

