# Supplementary material for: Guiding intensive care physicians’ communication and behavior towards bereaved relatives: study protocol for a cluster randomized controlled trial (COSMIC-EOL)
Source: Trials. 2018 Dec 22;19:698. doi: 10.1186/s13063-018-3084-7 (PMC6303988; doi:10.1186/s13063-018-3084-7)
Supplement: Supplementary file 1 — List of centers participating in the COSMIC-EOL trial. (DOCX 20 kb) [file 13063_2018_3084_MOESM1_ESM.docx]

**List of centers participating in the COSMIC-EOL trial**

| Name of the investigator | Hospital | City | Intensive Care Unit |
| --- | --- | --- | --- |
| 1. AZOULAY Elie | Saint Louis, AP-HP | Paris | Medical ICU |
| 2. CARIOU Alain | Cochin, AP-HP | Paris | Medical ICU |
| 3. CONSTANT Anne-Laure | HEGP, AP-HP | Paris | Cardio-surgical ICU |
| 4. DEMOULE Alexandre | La Pitié Salpétrière, AP-HP | Paris | Medical ICU |
| 5. CHAMPIGNEULLE Benoît | HEGP, AP-HP | Paris | Surgical ICU |
| 6. BOUADMA Lila | Bichat, AP-HP | Paris | Medical ICU |
| 7. FARTOUKH Muriel-Sarah | Tenon, AP-HP | Paris | General ICU |
| 8. GRIGORESCO Bénédicte | Beaujon, AP-HP | Clichy | Surgical ICU |
| 9. THUONG Marie | CH René Dubos | Pontoise | General ICU |
| 10. CHOUKROUN Gérald | CH Sud Francilien | Evry | General ICU |
| 11. SCHNELL David | CH d’Angoulême | Angoulême | General ICU |
| 12. GOLDGRAN-TOLEDANO Dany | GHI Montfermeil | Montfermeil | General ICU |
| 13. MERCERON Sybille | CH de Versailles | Le Chesnay | Medical ICU |
| 14 GUISSET Olivier | Saint André | Bordeaux | Medical ICU |
| 15. RENAULT Anne | CHU de la Cavale Blanche | Brest | Medical ICU |
| 16. LEBAS Eddy | CH Bretagne Atlantique | Vannes | Medical ICU |
| 17. ARGAUD Laurent | GH Edouard Herriot | Lyon | Medical ICU |
| 18. FLOCCARD Bernard | GH Edouard Herriot | Lyon | Surgical ICU |
| 19. PAPAZIAN Laurent | Hôpital Nord, AP-HM | Marseille | Medical ICU |
| 20. LEONE Marc | Hôpital Nord, AP-HM | Marseille | Surgical ICU |
| 21. RIGAUD Jean-Philippe | CH de Dieppe | Dieppe | General ICU |
| 22. REIGNIER Jean | CHU de Nantes, Hôtel Dieu | Nantes | Medical ICU |
| 23. JABER Samir | CHU Saint Eloi | Montpellier | Surgical ICU |
| 24. DELANNOY Pierre-Yves | Hôpital Chatilliez | Tourcoing | General ICU |
| 25. BOLLAERT Pierre-Edouard | CHU de Nancy- Hôpital Central | Nancy | Medical ICU |
| 26. PHILIPPON JOUVE Bénédicte | CH de Roanne | Roanne | General ICU |
| 27. VIQUESNEL Gérald | CHU de Caen Côte de Nacre | Caen | Surgical ICU |
| 28. TAMION Fabienne | CHU de Rouen | Rouen | Medical ICU |
| 29. RAMAKERS Michel | CH de Saint Lô | St Lo | General ICU |
| 30. LESIEUR Olivier | CH de La Rochelle | La Rochelle | General ICU |
| 31. KALFON Pierre | CH de Chartres | Chartres | General ICU |
| 32. CHOUQUER Renaud | CH d'Annecy | Annecy | General ICU |
| 33. ASEHNOUNE Karim | CHU de Nantes, Hôtel Dieu | Nantes | Surgical ICU |
| 34. FIANCETTE Maud | CHD Les Oudairies | La Roche sur Yon | Medical ICU |
| 35. TERZI Nicolas | CHU de Grenoble | Grenoble | Medical ICU |
| 36. LAUTRETTE Alexandre | CHU Gabriel Montpied | Clermont Ferrand | General ICU |
